# Evaluating the Benefits of Neuromuscular vs. Strength-Only Training in Athletes for ACL Re-Injury Prevention

**DOI:** 10.26502/josm.511500267

**Published:** 2026-04-27

**Authors:** Akshay Bharadwaj, Marcel P. Fraix, Devendra K. Agrawal

**Affiliations:** Department of Translational Research, Western University of Health Sciences, 309 E. Second Street, Pomona, California, USA

**Keywords:** Anterior Cruciate Ligament (ACL), ACL Reconstruction (ACLR), Biomechanical Outcomes, Knee Abduction Moment (KAM), Landing Mechanics, Neuromuscular Training (NMT), Re-injury Incidence, Return-to-sports (RTS), Secondary Prevention

## Abstract

Anterior cruciate ligament (ACL) injuries are one of the most prevalent injuries occurring in athletes. These injuries are highly prevalent in cutting sports such as basketball, football, and soccer. Additionally, there is a significant risk of re-injury for athletes who return to their sports without proper rehabilitation. Neuromuscular training, or NMT, is a more comprehensive form of rehabilitation focused on aspects such as plyometrics, agility training, and balance. The goal of this study is to compare the efficacy of NMT- focused rehabilitation to strength-only rehabilitation. This study was executed as a narrative review gathering various published research articles from Google Scholar and PubMed. The studies showed that NMT is strongly linked to lower re-injury rates compared to conventional rehabilitation programs. Arundale et al. (2018) reports a re-injury rate of 2.5% following an NMT-focused prevention program, while Paterno et al. (2014) reports a 29.5% re-injury rate following a strength-focused program. Additionally, specific improvements in biomechanical outcomes were noted, such as reduced knee abduction movement and improved hop test symmetry. The studies also highlighted predictors of risk of re-injury, such as younger age, female sex, and time-based return to sport as opposed to criteria-based return to sport. Overall, the conclusion is that NMT should be incorporated alongside a strength training program to produce the most optimal outcomes in athletes returning to their sport following an ACL injury.

## 1. Introduction

Anterior cruciate ligament (ACL) injuries are extremely common in sports, accounting for 100,000 to 200,000 injuries per year for athletes [1]. These injuries are common particularly for sports involving frequent pivoting and cutting actions such as basketball, football, and soccer [2]. The incidence of non-contact ACL injuries is significantly higher in female athletes compared to male athletes, at about 0.14 per 1000 player hours compared to 0.05 per 1000 player hours [3]. ACL injury risk can also be linked to multiple anatomical and physiological variances aside from the knee, including poor core stability, steeper posterior tibial slope, and weak hip abductor movement [1,4,5,6]. Interestingly, a genetic component may be involved; a significant non-modifiable risk factor for both male and female athletes is having a parent with a prior ACL injury [7].

Athletes who return to sport following ACL injury rehabilitation have a substantially increased risk of reinjury compared with athletes without a prior ACL injury [8,9]. Proper post-injury rehabilitation remains critical, as the risk of ACL tear persists even after surgical reconstruction [10]. Moreover, with inadequate rehabilitation, repetitive usage of the ligament can lead to detrimental life-long ailments, such as secondary arthritis [11].

Non-surgical interventions, particularly neuromuscular training (NMT), play an important role in maintaining knee stability. NMT broadly includes training components designed to enhance neuromuscular control and movement quality, such as plyometrics, balance and proprioceptive drills, and agility or movement pattern retraining [12-14]. Neuromuscular training may address deficits in biomechanics and neuromuscular control that contribute specifically to non-contact ACL injury mechanisms [15]. About 55% of all ACL injuries in team-ball sports occur through non-contact mechanisms such as twisting and turning, so incorporating NMT into rehabilitation addresses a large category of ACL injury risk [3]. Adding on, addressing the neuromuscular component targets the preventable aspects of ACL injury, which can overrule largely fixed factors such anatomical and hormonal risks [16]. Moreover, ACL injuries plague players for longer and may significantly reduce length of overall career, emphasizing the need for a thorough NMT-focused rehabilitation program [17].

Neuromuscular training also addresses the aspect of neuroplasticity that plays an important role in these injuries. When an athlete is injured, their brain shifts to focusing more on visual information rather than the physical signals from their own leg [18]. Thus, surgery alone is insufficient in protecting the leg; motor control from the brain is addressed and fixed through NMT.

Many studies have examined NMT in the context of primary ACL injury prevention, and have shown that there is an inverse relationship between NMT volume and ACL injury risk [19]. However, fewer investigations have focused on secondary prevention and ACL reinjury risk, particularly when comparing NMT-inclusive rehabilitation with strength-only training programs [10,20]. Additionally, many rehabilitation programs are not focused on high-risk groups and instead employ a uniform approach to treatment that does not address all individuals [21].

Our major goal of this article is to critical investigate the findings in the literature on a research question: “Does neuromuscular training (NMT) reduce the risk of ACL reinjury more effectively than strength training alone?” We hypothesized that athletes who participate in NMT will have lower rates of ACL re-injury or better functional outcomes compared to those undergoing strength training alone. Accordingly, (i) a critical evaluation was performed on the incidence of ACL reinjury in athletes who underwent strength-training only versus those who underwent a neuromuscular training program after initial ACL injury, (ii) a comparative analysis was done on the secondary biomechanical/functional outcomes between groups, such as documented landing mechanics, hop test performance, or return-to-sport timing, and (iii) key predictors (age, sex, sport type, graft type, time to return to sports) of reinjury and training effectiveness were examined.

## 2. Methods

This study was conducted as a narrative literature review of published studies with the primary focus of examining neuromuscular training–based rehabilitation following ACL injury. A targeted search of PubMed and Google Scholar was performed using keywords related to ACL reconstruction, neuromuscular training, reinjury, biomechanics, and return to sport. Studies reporting secondary ACL injury incidence, biomechanical or functional outcomes, neuromuscular training components, or predictors of reinjury were included. Data was extracted and summarized descriptively due to heterogeneity in study design and outcomes. Additional studies were selected to embellish related aspects of ACL injuries, such as psychological indicators of readiness, sex-specific risk factors, and more information on neuroplasticity. While the initial goal was to incorporate articles from the last 5 years, there was insufficient data on specific prevention programs comparing NMT vs strength-training, so the window was expanded to the previous 20 years. Keeping the wider window of the published reports in last 20 years ensures that foundational longitudinal studies are incorporated, giving weight to the arguments presented in the review.

## 3. Results

### 3.1 Reinjury Incidence Across Studies:

**Figure 1:**
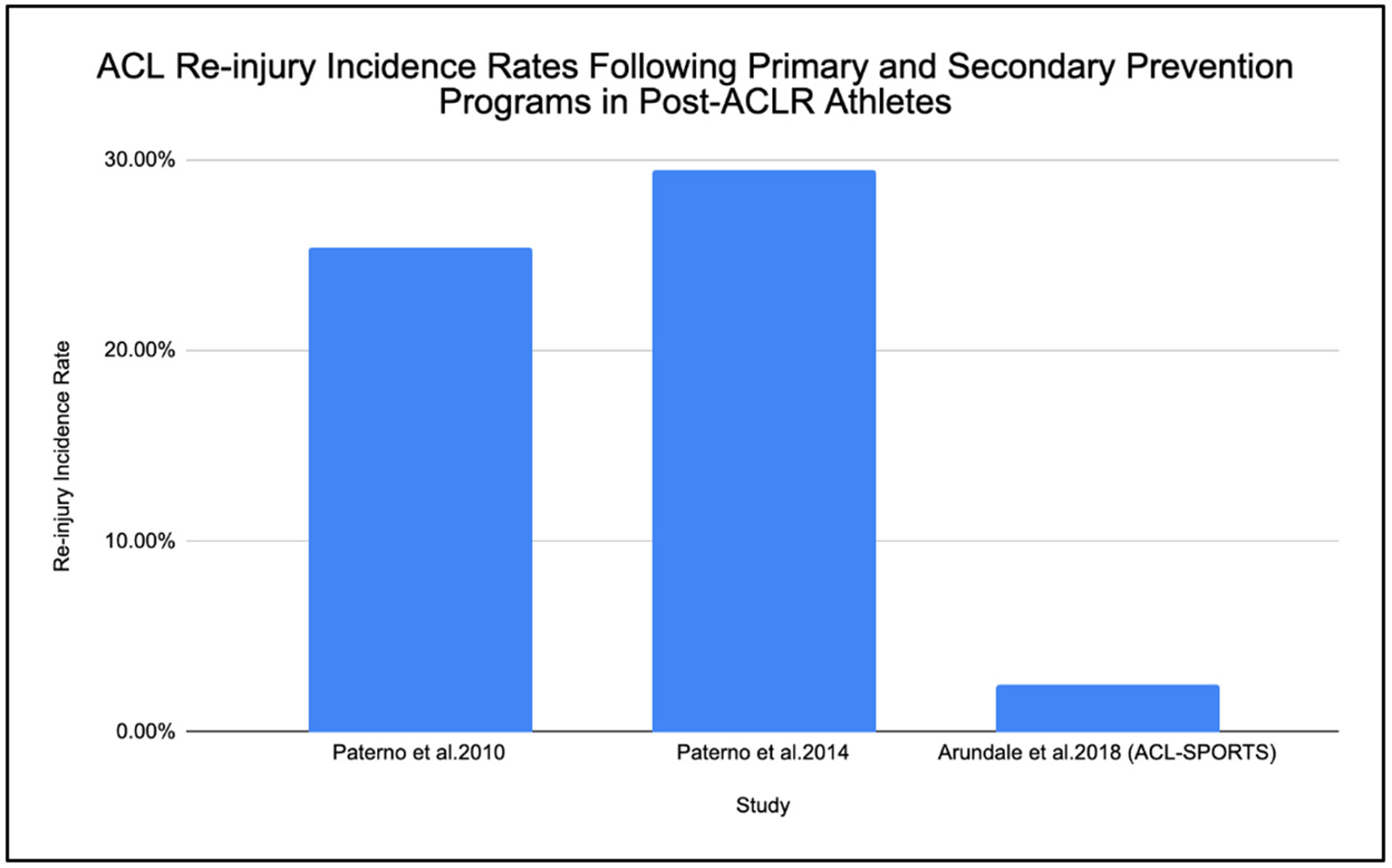
A bar diagram was constructed to visually compare reported ACL re-injury rates across 3 studies (Paterno et al. [8,9]; Arundale et al. [20]) evaluating neuromuscular training versus strength-based rehabilitation programs. Studies were selected based on reporting of ACL re-injury incidence following return to sport, inclusion of athletic populations, and description of rehabilitation strategies.

Three major studies were found in the literature with the evaluation of ACL re-injury rates comparing neuromuscular training versus strength-based rehabilitation programs (Figure 1). The Paterno et al. [8,9], which focused on a strength-based program, showed re-injury rates of 25.40% and 29.50%, respectively. The studies by Arundale et al. [20] focused on an NMT-incorporating program, and reported a re-injury rate of 2.5% (Figure 1 and Table 1).

### 3.2 Secondary Biomechanical and Functional Outcomes:

There are several reports in the literature comparing the secondary biomechanical and functional outcomes of post–ACL reconstruction rehabilitation (Table 1). Neuromuscular training programs demonstrate improved landing biomechanics, including reduced knee abduction moments (KAM), and superior functional performance on hop testing (reported with limb symmetry index (LSI)) compared with conventional or strength-focused rehabilitation.

### 3.3 Predictors of Reinjury and Training Effectiveness

Across included studies, several demographic and clinical factors were consistently associated with variation in second ACL injury risk and rehabilitation outcomes. Younger age was identified as a strong predictor of reinjury, with adolescent and young adult athletes demonstrating higher rates of second ACL injury following return to sport compared with older cohorts [8,9]. Female sex was also associated with increased reinjury incidence, particularly contralateral ACL injury [8,9]. Participation in pivoting or cutting sports, such as soccer and basketball, was similarly associated with higher reinjury risk relative to non-pivoting sports.

Timing and criteria for return to sport emerged as important predictors of outcomes. Earlier return to sport, particularly when based on time-based rather than criteria-based clearance, was associated with higher reported reinjury rates [9]. In contrast, studies incorporating structured, criteria-based return-to-sport progression, often alongside neuromuscular training–inclusive rehabilitation, demonstrated improved functional readiness at return, including higher hop test limb symmetry and lower observed reinjury incidence [20].

Neurocognition was also identified as a predictor injury, further emphasizing the need for NMT. Injuries in some studies were attributed to deficits in attention and visuospatial awareness, which can be remedied by incorporating these aspects into rehabilitation [7].

**Figure 2:**
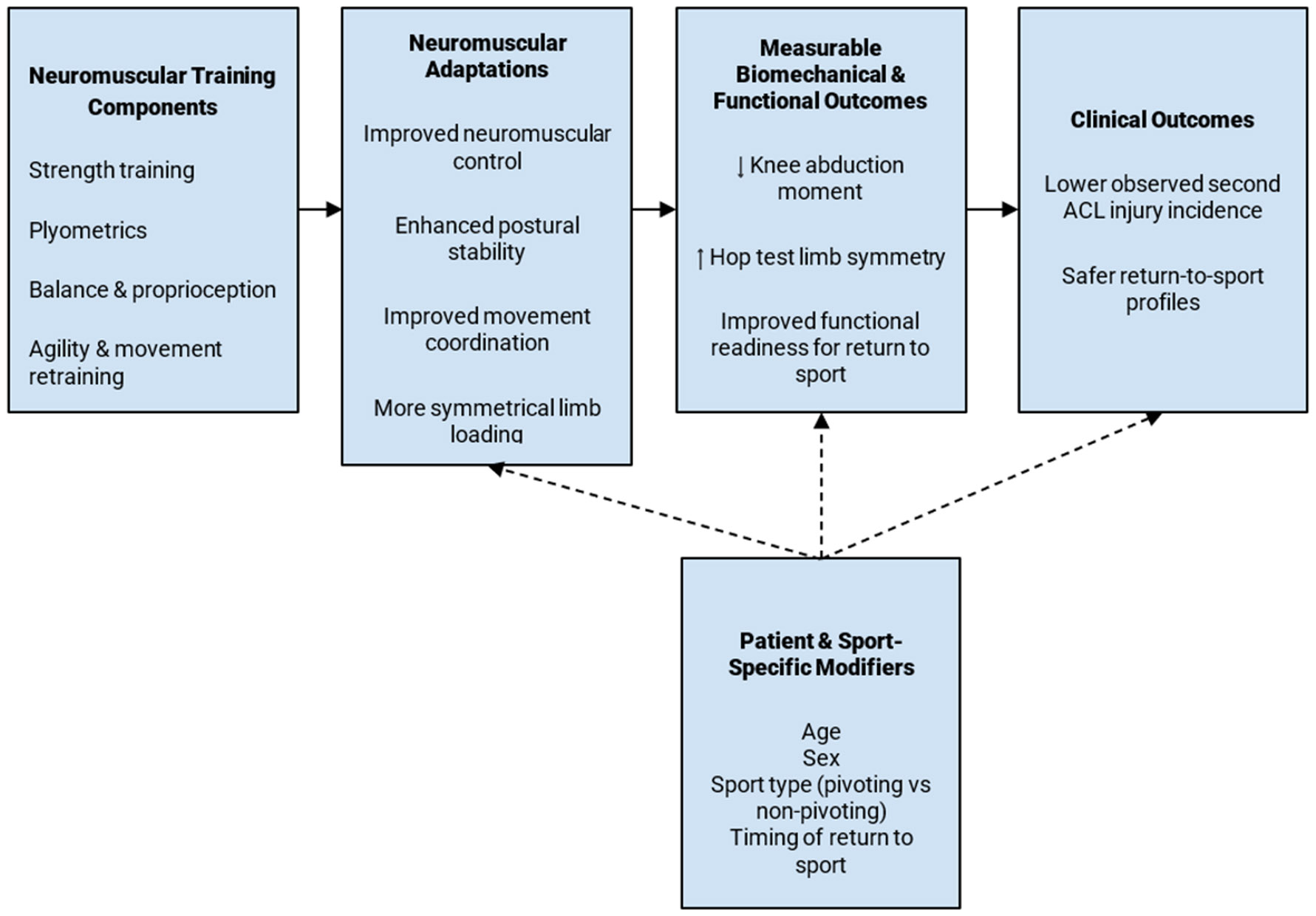
Conceptual framework summarizing the proposed relationship between neuromuscular training–inclusive rehabilitation and reduced risk of second anterior cruciate ligament (ACL) injury following return to sport. Neuromuscular training may lead to improvements in neuromuscular control and postural stability, which are associated with favorable biomechanical and functional outcomes such as reduced knee abduction moments, improved landing mechanics, and greater hop test limb symmetry. These improvements may contribute to lower observed reinjury incidence. Patient- and sport-specific factors, including age, sex, sport type, and timing of return to sport, may modify these relationships.

NMT’s utility is extremely valuable given that even under reduced training volumes, it is still shown to provide positive results. Thus, it should be considered a highly efficient form of rehabilitation, generating maximum protection with a minimal time investment [22] (Figure 2).

## 4. Discussion

A cross-study analysis demonstrates that neuromuscular training is associated with lower observed ACL reinjury incidence compared with conventional rehabilitation approaches. This association is supported by biomechanical evidence showing that NMT improves neuromuscular control, landing biomechanics, muscle activation patterns, and proprioception, all of which are factors known to influence non-contact ACL injury mechanisms [13, 14, 23]. More specifically, NMT aids in “pre-activating” the joint to prepare it for the harsh quick forces encountered during sport [24].

A comprehensive NMT approach must be followed, with different components targeting different aspects of fitness. A strength component will improve athletes’ in-game force capacity (Figure 2). Plyometric drills will help to simulate sport-specific jumping and landing. Movement pattern drills lower the risk of poor knee alignment with rapid random movements [25]. NMT provides an opportunity to deliver holistic training to athletes, ensuring that their bodies are well-rounded and suited for all conditions of their respective sports. It is crucial that these drills simulate the high-impact forces experienced during a real game scenario; for example, incorporating Forward Vertical Jump mimics the quadriceps-focused movements seen in basketball and football [26]. These drills should be started during adolescence, as this is when the nervous system is most malleable and responsive to the various balance and coordination challenges being administered [27].

The method of delivery also plays an important role in NMT. Rommers et al. [28] suggests that there should be an external focus, focusing on specific tasks such as landing, rather than an internal focus, focusing on specific body parts. This is the most optimal to improve motor control [28].

These studies emphasized other factors associated with re-injury incidence. Faster return to sport times, younger age, sports with fast pivoting/cutting movements and female athletes were more likely to face ACL re-injury. This highlights important implications for sports medicine teams, in that they should prioritize high-risk athletes and provide them with the opportunity to receive NMT. For example, female athletes have a wider pelvis, increased Q-angle, and narrower femoral notches, predisposing them to knee valgus and heightening the risk of injury [6]. These athletes should receive targeted treatment focusing on these deficits, focusing on their body-specific landing patterns and movement mechanics. Additional radiological screening can also be added to assess for anatomical risk factors, especially in children whose parents have a recurring history of injuries [29].

In addition, it can be hypothesized that NMT-focused training not only improves biomechanical outcomes, but can also boost confidence in athletes which plays a role in successful return to sport. Crampton et al shows that with an NMT-focused program for dancers, there were not only objective positive outcomes, such as increased triple-hop distance and decreased drill times, but also higher satisfaction ratings and higher athletic benefits assessed by the participants [30]. Furthermore, Laboute et al. [31] shows that athletes with high anxiety and low confidence following their initial injury have the lowest return-to-sport rate [31]. Mental status plays a crucial role in readiness; if an athlete feels that they can perform adequately and optimally following their rehabilitation, it is positively linked to a better performance [32]. NMT needs to include specific retraining of movement patterns and drills that builds athletes’ confidence in their knee, allowing them to perform at the highest level. Moreover, an emphasis should be made on starting NMT as early as possible in athletes. This allows them to attain a strong neuromuscular foundation that can continue to develop as they grow to a more elite level, while also developing their confidence as athletes [4].

A proper rehabilitation program is not always feasible. Extrinsic factors such as lack of access to equipment, poor-fitting training gear, and low quality playing surfaces make it harder to execute these programs to the extent that is required [33]. When designing NMT-focused programs, attention must be given to these factors and designed in a way that is tailored to the school’s capabilities. However, developments are being made in providing NMT in a remote, video-based manner, addressing the disparities in treatment that some schools might face [34].

Studies also state the importance of compliance. These programs must be performed multiple times a week in order to be effective, and lack of adherence is shown to reverse positive outcomes, even with a well designed program [7, 35, 36]. The lack of adherence typically stems from lack of knowledge about NMT and its utility. It is something that many student athletes are not explicitly taught about, which may lead to an inability to adhere to training [37]. Effective instructional guidance regarding NMT is necessary to facilitate consistent adherence, which will optimize the efficacy of the training and promote success [38].

There are still limitations in the current realm of NMT studies. Many publications did not have normalized data to compare strength-only versus NMT-centered rehabilitation programs. Additionally, there were not many publications focused on the aforementioned high-risk groups and the impact of NMT on them. More research must be done on NMT and its efficacy, particularly how it can affect modifiable risk factors and how it can improve outcomes in high-risk athletes.

Research should also focus on the incidence of “successful” return to sport following NMT. There is a distinction between returning to playing generally and returning to playing at a competitive level [39]. Many athletes are not able to return to the same level at which they once were, and perhaps NMT can help bridge the gap between these levels [40].

## 5. Conclusion

Studies showed that ACL re-injury incidence was lower in neuromuscular training–focused rehabilitation programs compared with conventional or strength-only programs. Additionally, the literature showed that NMT improved various biomechanical outcomes following injury, such as decreased knee abduction movement and improved hop-test performance. Studies also identified multiple high-risk groups for ACL re-injury, such as female athletes, younger athletes, and participation in “cutting sports.” Therefore, athletes recovering from ACL injury should ideally participate in a structured neuromuscular training program in addition to standard strength training to minimize risk of reinjury. An ideal NMT program not only improves physical outcomes but should bolster confidence in young athletes, giving them the mental fortitude needed to perform at their highest capabilities.

## Figures and Tables

**Table 1:** Comparison of secondary biomechanical and functional outcomes reported in studies of post–ACL reconstruction rehabilitation. The outcomes are reported as directional changes and % limb symmetry index (LSI), as described in each study. KAM, knee abduction moments; NMT, neuromuscular training; RTS, return to sports.

Study	Rehab Classification	Peak Knee Abduction Moment	Hop Test Performance (LSI %)	Time to RTS (months)	Key Findings
Paterno et al. [8,9]	Conventional / mixed rehab	↑ Elevated KAM in reinjured athletes	Not reported	Not reported	Abnormal landing biomechanics associated with second ACL injury
Myer et al. [12]	Neuromuscular training	↓ KAM after NMT	↑ Improved symmetry (>90%)	Not reported	NMT improved landing mechanics and neuromuscular control
Wordeman and Hewett [10,13,14]	Neuromuscular training	↓ Knee valgus and KAM	Not reported	Not reported	NMT reduced high-risk landing patterns
Arundale et al. (ACL-SPORTS) [20]	Secondary prevention program with NMT elements	Not directly reported	↑ High hop symmetry (≥95%)	~7–9 months	Functional readiness improved with criteria-based RTS

## 6. Key Points

- Athletes returning to sport after an ACL injury face a higher risk of ACL re-injury compared to those who have not had an ACL injury.
- Rehabilitation programs focusing on neuromuscular training show lower ACL re-injury rates compared to programs that focus on strength only.
- NMT prevents re-injury by minimizing injurious landing patterns, specifically by decreasing knee abduction movement.
- Athletes in NMT-focused programs perform better on functional outcome tests, specifically demonstrating higher limb symmetry indices on hop tests.
- The most optimal rehabilitation programs provide a combination of NMT and strength focused objectives in order to be multifaceted and treat various movement issues.
- Younger age, female sex, and time-based return to sport are among the strongest predictors of ACL re-injury occurrence.
- Proper ACL rehabilitation is important not only for optimal sport performance, but for preventing long-term secondary health issues such as arthritis.
- More research is warranted on the effect of NMT on high-risk athlete groups, specifically how their performance and re-injury rates are affected.
